# Resilience, pain, and health-related quality of life in gynecological patients undergoing surgery for benign and malignant conditions: a 12-month follow-up study

**DOI:** 10.1186/s12905-022-01923-7

**Published:** 2022-08-16

**Authors:** Siiri Isokääntä, Ulla-Maija Ruohoaho, Maarit Anttila, Hannu Kokki, Harri Sintonen, Petri Toroi, Merja Kokki

**Affiliations:** 1 Department of Anesthesia and Operative Services, Kuopio University Hospital (KYS), Puijonlaaksontie 2, PO Box 100, 70029 Kuopio, Finland; 2 School of Medicine, Faculty of Health Sciences, University of Eastern Finland, Kuopio, Finland; 3 Department of Gynecology, Kuopio University Hospital, Kuopio, Finland; 4 Department of Public Health, University of Helsinki, Helsinki, Finland

**Keywords:** Resilience, Pain, Quality of life, Gynecologic surgery, Anxiety, Depression, Oxycodone

## Abstract

**Background:**

Gynecological surgery has many impacts on women’s physical and mental health, and efforts to improve recovery from surgery are constantly under evaluation. Resilience is an ability to overcome stressors and adversities, such as traumas and surgeries. This study aimed to explore patients’ resilience and psychological symptoms in relation to recovery, health-related quality of life (HRQoL), and pain one year after gynecological surgery.

**Methods:**

In a prospective cohort study, we enrolled consecutive elective gynecologic surgery patients who completed questionnaires before and at one year after surgery: the Resilience Scale-25, the 15D instrument of HRQoL (15D), the Life Satisfaction Scale-4, and the Hospital Anxiety and Depression Scale. Their mean 15D scores were compared to those of an age-matched sample of women from the general Finnish population (n = 2743).

**Results:**

We enrolled 271 women who underwent gynecological surgery due to benign (n = 190) and malignant (n = 81) diagnoses. Resilience was equally high in women with benign and malignant diagnoses at both time points. Higher resilience associated with less pain, analgesic use, and better pain relief from the use of pain medication at 12 months after surgery. Pain intensity was similar in the two groups, but patients with benign diseases had less pain at 12 months than before surgery. Before surgery, patients’ HRQoL was worse than that of the general population, but at 12 months the mean HRQoL of patients with benign diseases had improved to the same level as that in the general population but had decreased further in patients with malignant diseases. Anxiety was higher and life satisfaction was lower in patients with malignant diseases before surgery. At 12 months, anxiety had decreased in both groups, and life satisfaction had increased in patients with malignant diseases. Depression was similarly low in both groups and time points.

**Conclusions:**

Resilience correlated with less pain one year after surgery. After surgery, HRQoL improved in patients with benign diseases but deteriorated in patients with malignant diseases. Patients with low resilience should be identified during preoperative evaluation, and health care professionals should give these patients psychological support to enhance their resilience.

*Trial Registration* ClinicalTrials.gov; registered October 29, 2019; identifier: NCT04142203; retrospectively registered.

## Background

Gynecologic surgery is a common procedure [1] that has many effects on women’s physical and mental health [2, 3]. In Finland, with a population of 5.5 million, 25,000 women undergo gynecological surgery annually. Most gynecologic surgeries are performed because of benign diagnoses. Common benign indications for gynecologic surgery are myomas, uterine prolapses, and menorrhagia [1]. Frequent malignant diagnoses include uterine, ovarian, and cervical cancer [4]. Regardless of the diagnosis, admission to the hospital and the surgery itself can cause substantial anxiety [5], but it often abates over time [6, 7]. However, some patients, especially those with low resilience, are at risk of persistent anxiety and depression [5, 7, 8].

Resilience is an ability to adapt to adversity, such as surgeries, health-related issues, or other sources of stress in life [9]. Most people are sufficiently resilient, but resilience skills in those with less adaptive capacities can be learned and enhanced [9, 10]. Resilience has gained increasing research interest in the medical setting due to its associations with a better recovery, enhanced quality of life [11, 12], and functional status [13] and fewer psychological symptoms [14, 15] and less pain interference after surgery [15, 16]. A meta-analysis showed a cautious positive effect at improving resilience and other health outcomes [17].

Some data indicate that the health-related quality of life (HRQoL) often improves after gynecologic surgery in both benign and malignant conditions [2, 7, 18]. As cancer treatments and survival improve and treatment costs soar, HRQoL has become an important outcome parameter when weighing treatment options, but it is also important in benign gynecological diagnoses [2]. However, there are few data on how resilience is associated with the outcome after surgery and whether the diagnosis may affect this potential association.

The aim of this prospective, one-year follow-up study was to investigate how gynecologic surgery in both benign and malignant conditions may affect psychological factors, including resilience, anxiety, and depression, and how they relate to recovery, HRQoL and pain. We also evaluated how the HRQoL of surgical patients compared with that of the general population. The primary outcome measures were HRQoL and pain intensity one year after surgery. We hypothesized that higher resilience before surgery is associated with lower anxiety and depression, less pain, less need for pain medication, and a better HRQoL one year after surgery.

## Methods

### Study design and participants

This study was a prospective cohort study of gynecological patients undergoing surgery for benign and malignant conditions in Kuopio University Hospital (KUH), Kuopio, Finland, between June 2017 and October 2019. It was a part of a larger study [19] that received the study protocol amendment approval of The Research Ethics Committee of the Northern Savo Hospital District, Kuopio, Finland (No. 73/2017; February 7, 2017). No data on these patients has been previously published. Institutional approval (No. 112/2016) was updated (No. 27/2019) after the new regulation of the European Union on the protection of natural persons with regard to the processing of personal data (2016/679). This study complies with the principles of the Declaration of Helsinki.

Patients were enrolled during their preoperative gynecologist’s visit or, if no preoperative visit to the hospital occurred, on the morning when they arrived for surgery. Patients were given oral and written information about the study and enough time to consider their participation. They were also encouraged to contact the researchers if they had anything to discuss about the study. Patients who agreed to participate in the study gave their written informed consent. Eligible participants were 18 years old or older, under consideration for elective gynecology surgery in KUH, and able to understand the study protocol and complete questionnaires in the Finnish language. We did not enroll patients with serious mental illnesses, such as schizophrenia, bipolar disorder or major depression, low cognitive functions, e.g., dementia, or alcohol or drug addiction.

We recruited consecutive elective gynecologic surgery patients from KUH during a two-year period and compared the outcome based on whether the diagnosis was benign or malignant. This was expected to give a representative sample of both groups and minimize selection bias. No formal study size calculations were performed, but approximately 300 patients were expected to give a representative sample of women undergoing surgery with both benign and malignant diseases.

The questionnaires comprised background data, questions about pain and pain medication, and questionnaires about resilience, life satisfaction, anxiety, depression, and HRQoL. The questionnaires were completed before surgery and at 12 months after surgery. The questionnaires were web-based, but hard copies were available based on patient preferences. In these cases, the 12-month follow-up questionnaires had a prepaid envelope for return to the researchers.

### Data collection

Patient data (Table 1) were collected from the KUH electronic medical records (EMRs, Oberon® and Uranus®, CGI, Helsinki, Finland) along with operative (Table 2), pain and pain medication data (Table 3). Hospital readmissions within the first 28 postoperative days, reoperations, and new operations within the first 12 months after the surgery were retrieved from the EMRs. Mortality was checked in March 2021; the entire follow-up time for all patients was 36 months (range 17–45). One-year mortality was calculated from the surgery date. Pain intensity and pain relief were assessed with an 11-point numerical rating scale (NRS) ranging between 0 (no pain/no pain relief) and 10 (most pain/complete pain relief). The hospital stay duration was calculated from patient arrival at the hospital to when the patient was discharged from the KUH.

**Table 1:** Patient characteristics at baseline. Data are mean (SD) or number of cases (%)

Variable	All participants (N = 271)	Benign disease (n = 190)	Malignant disease (n = 81)	*p* value
*Age (years)*	56.6 (14.4)	53.5 (14.2)	64.1 (11.8)	< 0.001
*Height (cm)*	163 (6)	163 (6)	163 (5)	0.38
*Weight (kg)*	75 (16)	74 (15)	78 (18)	0.067
*BMI (kg/m*^*2*^*)*	28.2 (5.7)	27.6 (5.1)	29.6 (6.8)	0.018
*Number of comorbidities, ICD-10*	2 (2)	2 (2)	3 (2)	0.11
I00-I99: Cardiovascular	123 (45%)	80 (42%)	43 (53%)	0.092
E00-E90: Endocrine and metabolic	105 (39%)	66 (35%)	39 (48%)	0.097
M00-M99: Musculoskeletal	62 (23%)	40 (21%)	22 (27%)	0.43
G00-G99: Nervous system	45 (17%)	31 (16%)	14 (17%)	0.91
J00-J99: Respiratory	44 (16%)	30 (16%)	14 (17%)	0.85
Other: C00-C48/D50-D89/F00-F99/H00-H59/H60-H95/K00-K99/L00-L99/N00-N99	35/10/18/14/6/34/12/21	22/8/13/5/2/23/8/18	13/2/5/9/4/11/4/3	
*Number of regular medicines*	3 (3)	3 (3)	4 (3)	0.02
*Smoking*				
Current smoker (yes)	32 (12%)	27 (14%)	5 (6%)	0.063
Cigarettes/day	9 (5)	9 (5)	11 (7)	0.54
12 months after surgery (yes)	20 (8%)	16 (10%)	4 (6%)	0.32
*ASA class (1/2/3/4)*	55/143/68/5	45/105/38/2	10/38/30/3	0.001
*Mortality*				
12 months	5 (1.8%)	0	5 (6.2%)	0.002
Entire follow-up	10 (3.7%)	1 (0.5%)	9 (11.1%)	< 0.001
Time from surgery to death (months)	13.6 (6.9)	21	12.8 (6.7)	0.40
Follow-up time (months)	34.6 (6.3)	34.6 (6.2)	34.6 (6.6)	0.98

*BMI* body mass index, *ASA* The American Society of Anesthesiologists’ Physical Status Classification System

**Table 2:** Operative and hospital stay information. Data are mean (SD) or number of cases (%)

Variable	All participants (N = 271)	Benign disease (n = 190)	Malignant disease (n = 81)	*p* value
*Surgery procedure, n (%)*				
Major cancer surgery	73 (27%)	–	73 (90%)	
Minor cancer surgery	8 (3%)	–	8 (10%)	
Major other surgery	55 (20%)	55 (29%)	–	
Intermediate other surgery	91 (34%)	91 (48%)	–	
Minor other surgery	44 (16%)	44 (23%)	–	
*Surgery technique, n (%)*				< 0.001
Laparotomy	22 (8%)	5 (3%)	17 (21%)	
Laparoscopy	155 (57%)	97 (51%)	58 (72%)	
Combined vaginal and laparoscopic surgery	20 (8%)	19 (10%)	1 (1%)	
Vaginal surgery	63 (23%)	63 (33%)	–	
Other	11 (4%)	6 (3%)	5 (6%)	
Genital ± perianal resection	8 (3%)	3 (1.5%)	5 (6%)	
Transvaginal tape	3 (1%)	3 (1.5%)	–	
*Surgery duration (min)*	152 min (98)	114 min (60)	242 min (111)	< 0.001
*Surgery process, n (%)*				< 0.001
Day surgery	38 (14%)	38 (20%)	0 (0%)	
23-h surgery	82 (30%)	80 (42%)	2 (2%)	
Inpatient surgery	151 (56%)	72 (38%)	79 (98%)	
*Hospital stay duration (h)*	50 h (59)	30 h (25)	96 h (86)	< 0.001
*PACU stay duration (h)*	10 h (7)	12 h (8)	5 h (4)	< 0.001
*Discharge from PACU, n (%)*				< 0.001
Home	120 (44%)	118 (62%)	2 (2%)	
KUH ward	151 (56%)	72 (38%)	79 (98%)	
*Discharge after KUH ward, n (%)*				
Home	148 (98%)	72 (100%)	76 (96%)	
Other hospital	3 (2%)	-	3 (4%)	
*Admission/procedure after discharge, n (%)*				
Readmission during the first 28 days	18 (7%)	9 (5%)	9 (11%)	0.054
Reoperations within 12 months	12 (5%)	5 (3%)	7 (11%)	0.047
Other operation within 12 months	19 (8%)	14 (8%)	5 (8%)	0.72

*PACU* postanesthesia care unit, *KUH* Kuopio University Hospital

**Table 3:** Pain and pain medication data. Data are mean (SD) or number of cases (%)

Variable	All participants (N = 271)	Benign disease (n = 190)	Malignant disease (n = 81)	*p* value
*Preoperative pain and analgesics*				
Had pain before surgery, n (%)	116 (43%)	82 (43%)	34 (42%)	0.86
Most pain at rest, NRS 0–10	1.5 (2.3)	1.6 (2.4)	1.4 (2.2)	0.47
Most pain at movement, NRS 0–10	1.9 (2.6)	1.9 (2.7)	1.7 (2.5)	0.62
Had preoperative pain medication, n (%)	169 (62%)	121 (64%)	48 (59%)	0.49
Paracetamol	117 (43%)	81 (42%)	36 (44%)	0.78
NSAID	108 (40%)	83 (44%)	25 (31%)	0.048
Opioid	15 (6%)	12 (6%)	3 (4%)	0.39
Adjuvant^a^	10 (4%)	7 (4%)	3 (4%)	0.99
Pain relief achieved, NRS 0–10	6.5 (3.4)	6.5 (3.5)	6.5 (3.0)	0.98
Analgesics’ adverse effects, n (%)	18 (7%)	12 (6%)	6 (7%)	**0.77**
*Perioperative pain and analgesics*				
Pain during the first 24 h after surgery				
Least, NRS 0–10	0.2 (0.5)	0.2 (0.6)	0.1 (0.3)	0.003
Most, NRS 0–10	3.9 (2.3)	3.8 (2.3)	4.2 (2.3)	0.16
Average, NRS 0–10	1.7 (1.2)	1.7 (1.2)	1.6 (1.3)	0.83
Pain at PACU discharge, NRS 0–10	1.0 (1.0)	1.0 (1.0)	0.9 (1.0)	0.23
Perioperative pain medication, n (%)				
Paracetamol	267 (99%)	188 (99%)	79 (98%)	0.38
NSAID	189 (70%)	150 (79%)	39 (48%)	< 0.001
Opioid	267 (99%)	186 (98%)	81 (100%)	0.12
S-Ketamine	47 (17%)	24 (13%)	23 (28%)	0.002
Epidural infusion	42 (16%)	19 (10%)	23 (28%)	< 0.001
*Pain and analgesics at 12 months after surgery*				
Had pain at 12 months, n (%)	107 (44%)	70 (41%)	37 (52%)	0.11
Surgery-related pain	31 (13%)	18 (11%)	13 (18%)	0.21
Most pain at rest, NRS 0–10	1.0 (1.9)	1 (2.0)	1.0 (1.7)	0.74
Most pain at movement, NRS 0–10	1.0 (1.9)	1 (1.8)	1.2 (1.9)	0.23
Pain medication at 12 months, n (%)	114 (47%)	74 (43%)	40 (56%)	0.064
Paracetamol	85 (35%)	55 (32%)	30 (42%)	0.13
NSAID	63 (26%)	47 (27%)	16 (23%)	0.42
Opioid	12 (5%)	6 (4%)	6 (8%)	0.11
Adjuvant^b^	10 (4%)	4 (2%)	6 (8%)	0.03
Pain relief achieved (NRS 0–10)	4.5 (3.9)	3.7 (3.9)	6.5 (3.3)	< 0.001
Analgesics’ adverse effects, n (%)	22 (9%)	15 (9%)	7 (10%)	0.67

*NRS* numeral rating scale (0 = No pain/no pain relief, 10 = Most pain/complete pain relief), *NSAID* non-steroidal anti-inflammatory drug, *PACU* postanesthesia care unit

^a^Adjuvant analgesics: amitriptyline, gabapentinoids, pitofenone, trimipramine, triptans

^b^Adjuvant analgesics: amitriptyline, carbamazepine, gabapentinoids, triptans, venlafaxine

Resilience was assessed with the *Resilience Scale-25* (RS-25, [20, 21]). It is a self-administered instrument that has 25 statements that measure subjective and interpersonal protective resources aiding in adaptation to adversities, and they are scored on a 7-point Likert scale from 1 (strongly disagree) to 7 (strongly agree). Total scores vary from 25 to 175 and they are classified from very low resilience (scores from 25 to 100), low resilience (scores from 101 to 115), moderately low resilience (scores from 116 to 130), moderate resilience (scores from 131 to 145), moderately high resilience (scores from 146 to 160), to high resilience (scores from 161 to 175). The dichotomized resilience scores comprised low resilience (scores from 25 to 130) and moderate or high resilience (scores from 131–175) [21].

Health-related quality of life was measured with the *15D instrument* [22]. It is a generic, self-administered instrument with 15 dimensions comprised of mobility, vision, hearing, breathing, sleeping, eating, speech (communication), excretion, usual activities, mental function, discomfort and symptoms, depression, distress, vitality, and sexual activity with five levels of severity on each to choose from. The 15D can be used both as a profile and a single index score measure. The single index score (15D score), representing the overall HRQoL on a 0–1 scale (1 = full health, 0 = being dead) and the dimension level values, reflecting the goodness of the levels relative to no problems on the dimension (= 1) and to being dead (= 0), are calculated from the questionnaire by using a set of population-based preference or utility weights. The minimum clinically important change or difference in the total 15D score is ≥ 0.015 [23].

We compared the HRQoL of gynecologic surgery patients to a sample from the general Finnish population. The data were derived from the National Health 2011 Health Examination Survey representing the adult population (18 years and older) [24]. For the comparison those women were selected from the Survey (n = 2743), who were in the age range of patients (18–84 years). This sample was weighted to reflect the age distribution of the patients.

Life satisfaction was assessed with the *Life Satisfaction Scale-4* (LS-4, [25]). The LS-4 is a self-assessment measure that has four items measuring interest and happiness in life, ease of living, and loneliness. The four items are scored on a 5-point Likert scale of decreasing life satisfaction. Total scores vary from 4 to 20. The respondent is satisfied with life with scores from 4 to 6, slightly dissatisfied with scores from 7 to 11, and dissatisfied with life with scores from 12 to 20. We dichotomized the scores to “satisfied with life” (scores from 4 to 11) and “dissatisfied with life” (scores from 12 to 20).

Anxiety and depressive symptoms were measured with the *Hospital Anxiety and Depression Scale* (HADS, [26, 27]). It is a self-report measure with 14 items, each is scored from 0 to 3 of increasing anxiety or depression. Even-numbered questions evaluate depression, and odd-numbered questions evaluate anxiety symptoms. Anxiety and depression scores were calculated separately and grouped into non-cases (scores from 0 to 7), mild anxiety or depression (scores from 8 to 10), moderate anxiety or depression (scores from 11 to 14), and severe anxiety or depression (scores from 15 to 21). The dichotomized data comprised no to mild symptoms (scores from 0 to 10) and moderate to severe anxiety or depression (scores from 11 to 21).

Primary outcomes were HRQoL and pain intensity one year after surgery. Secondary outcomes were anxiety, depression, and life satisfaction one year after surgery. Resilience before surgery was hypothesized to be a predictor of good HRQoL and low pain intensity.

### Statistics

The data were entered and analyzed with SPSS software version 27 (IBM SPSS Statistics, International Business Machines Corporation, Armonk, NY, USA). We compared the means of continuous variables between the groups using independent-samples t-tests and distributions (medians) with Mann–Whitney U tests, and the categorical variables were compared using χ^2^ or Fisher’s exact test. Paired-samples t-tests and Wilcoxon tests were chosen for continuous variables of dependent samples, and McNemar tests were chosen for dichotomized variables. Correlations between variables were evaluated with Pearson’s r: values indicating a weak correlation were between − 0.29 and + 0.29, moderate between − 0.49 and − 0.3 or 0.3 and 0.49, strong between − 0.89 and − 0.5 or 0.5 and 0.89, and very strong between − 1.0 and − 0.9 or 0.9 and 1.0. Cronbach’s alpha was calculated to measure the internal consistency for each questionnaire. The data are displayed as the number of cases, mean (SD), median (min–max), 95% confidence interval (CI), and mean difference, as appropriate. A *p* value of < 0.05 was considered statistically significant.

## Results

**Fig. 1:**
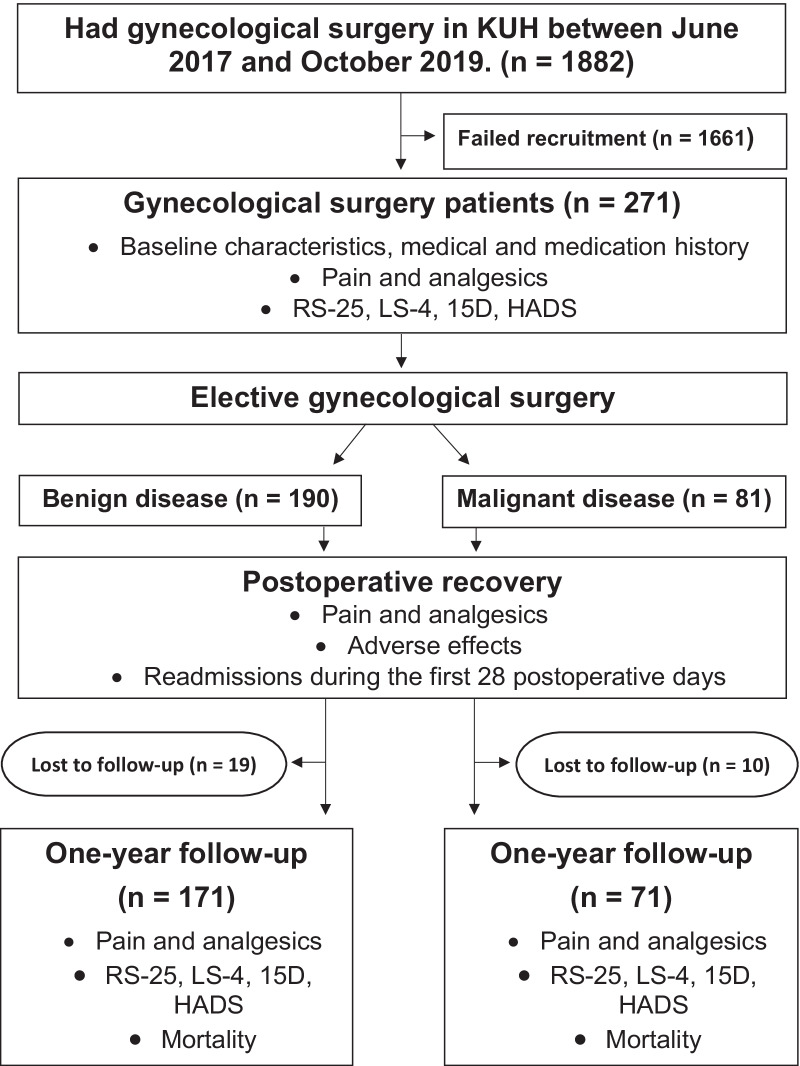
Flow chart of participants. KUH = Kuopio University Hospital; RS-25 = Resilience Scale-25; LS-4 = Life Satisfaction Scale-4; 15D = 15D Instrument of Health-related Quality of Life; HADS = Hospital Anxiety and Depression Scale

The patient baseline characteristics are listed in Table 1, operative and hospital stay data in Table 2, pain and pain medication data in Table 3, and data on psychological variables, HRQoL and life satisfaction in Table 4. Our sample consisted of 271 women before surgery and 242 at 12 months (response rate 89%). The response rate was equally high in patients with benign diseases, 171 out of 190 (90%), and in patients with malignant diseases, 71 out of 81 (87%). Figure 1 shows the flow chart. The internal consistency for RS-25 in our sample was excellent (Cronbach’s alpha = 0.933), for the 15 D instrument and HADS it was good (15D: 0.842; HADS: 0.863), and for the LS-4 it was acceptable (Cronbach’s alpha = 0.712).

**Table 4:** Resilience, health-related quality of life, life satisfaction, anxiety, and depression scores

Variable	Resilience (RS-25) Scale 25–175	Health-related quality of life (15D) Scale 0.0–1.0	Life satisfaction (LS-4) Scale 4–20	Anxiety (HADS) Scale 0–21	Depression (HADS) Scale 0–21
*Benign disease before surgery (n = 190)*					
Mean (SD)	141.7 (18.8)	0.902 (0.082)	8.1 (2.9)	5.9 (3.2)	2.8 (2.8)
Dichotomized^a^ (n)	141/44	NA	160/24	166/17	179/4
*Benign disease at 12 months after surgery (n = 171)*					
Mean (SD)	142.8 (21.9)	0.920 (0.086)	7.8 (2.7)	5.2 (2.8)	2.9 (2.8)
Dichotomized^a^ (n)	131/40	NA	152/19	161/10	168/3
Benign before surgery versus at 12 months: mean difference [95%CI]; *p* value	− 0.6 [− 3.7, 2.4]; *p* = 0.67	− 0.012 [− 0.026, 0.002]; *p* = 0.09	0.2 [− 0.2, 0.6]; *p* = 0.25	0.5 [0.1, 1.0]; *p* = 0.016	− 0.1 [− 0.5, 0.2]; *p* = 0.56
*Malignant disease before surgery (n = 81)*					
Mean (SD)	141.7 (18.8)	0.896 (0.085)	9.1 (3.3)	7.0 (3.5)	3.4 (2.7)
Dichotomized^a^ (n)	59/19	NA	64/16	67/13	80/0
Benign versus Malignant before surgery: mean difference [95%CI]; *p* value	0.041 [− 4.9, 4.9]; *p* = 0.99	0.007 [− 0.021, 0.034]; *p* = 0.64	− 1.0 [− 0.2, − 1.8]; *p* = 0.011	− 1.2 [− 0.3, − 2.0]; *p* = 0.008	− 0.6 [− 1.3, 0.2]; *p* = 0.12
*Malignant disease at 12 months after surgery (n = 71)*					
Mean (SD)	143.1 (22.6)	0.878 (0.099)	8.2 (3.1)	5.2 (3.1)	3.4 (3.4)
Dichotomized^a^ (n)	54/15	NA	64/7	68/3	69/2
Malignant before surgery vs. at 12 months: mean difference [95%CI]; *p* value	− 1.4 [− 5.2, 2.4]; *p* = 0.46	0.016 [− 0.006, 0.038]; *p* = 0.15	0.7 [0.1, 1.4]; *p* = 0.023	1.7 [1.1, 2.4]; *p* = 0.001	− 0.1 [− 0.8, 0.6]; *p* = 0.75
Benign vs. Malignant at 12 months: mean difference [95%CI]; *p* value	− 0.4 [− 6.6, 5.8]; *p* = 0.91	0.042 [0.007, 0.076]; *p* = 0.02	− 0.4 [− 1,2, 0.4]; *p* = 0.34	0.01 [− 0.8, 0.8]; *p* = 0.97	− 0.6 [− 1.5, 0.3]; *p* = 0.22

^a^Dichotomized: RS-25: > 130/ ≤ 130; LS-4: 4–11/12–20; Anxiety: 0–10/11–21; Depression: 0–10/11–21

Gynecological cancers (n = 81) comprised uterine (n = 50), ovarian (n = 17), vulvar (n = 6), cervical (n = 5) and tubal cancer (n = 2), and unknown origin (n = 1). Common benign diagnoses (n = 190) included gynecological prolapses (n = 59), ovarian tumors and cysts (n = 40), uterine tumors (n = 31), endometriosis (n = 18), and menstrual disorders, including menorrhagia, postmenopausal bleeding, and pain (n = 12).

The success rate of the short-stay surgery process was moderate; in the day surgery and 23-h surgery model, 85% (n = 120) of patients were discharged from the postanesthesia care unit (PACU) on the same day or within 24 h as planned, and 21 patients were admitted to the ward. Of the patients scheduled for inpatient surgery 129 were admitted to the ward as planned, one woman with extensive cancer surgery and major intraoperative bleeding was admitted to the intensive care unit after the surgery.

Perioperative adverse events were common, but most were mild. One hundred nine patients (40%) had at least one adverse event, and the most common were nausea and vomiting (n = 93), dizziness (n = 18), itch (n = 7), arrhythmia (n = 5), and high or low blood pressure (n = 5). Other complications (n = 6) included acute coronary syndrome (n = 1), falling and fracture (n = 1), vocal cord hematoma (n = 1), hemorrhage (n = 1), anaphylaxis (n = 1), and unintended cutting during surgery (n = 1).

One-year mortality was 1.8% (n = 5), and mortality for a mean of 36 months follow-up time was 3.7% (n = 10).

### Primary outcome measures: health-related quality of life and pain

The questionnaire total scores and number of observations in the dichotomized questionnaire scores are listed in Table 4.

**Fig. 2:**
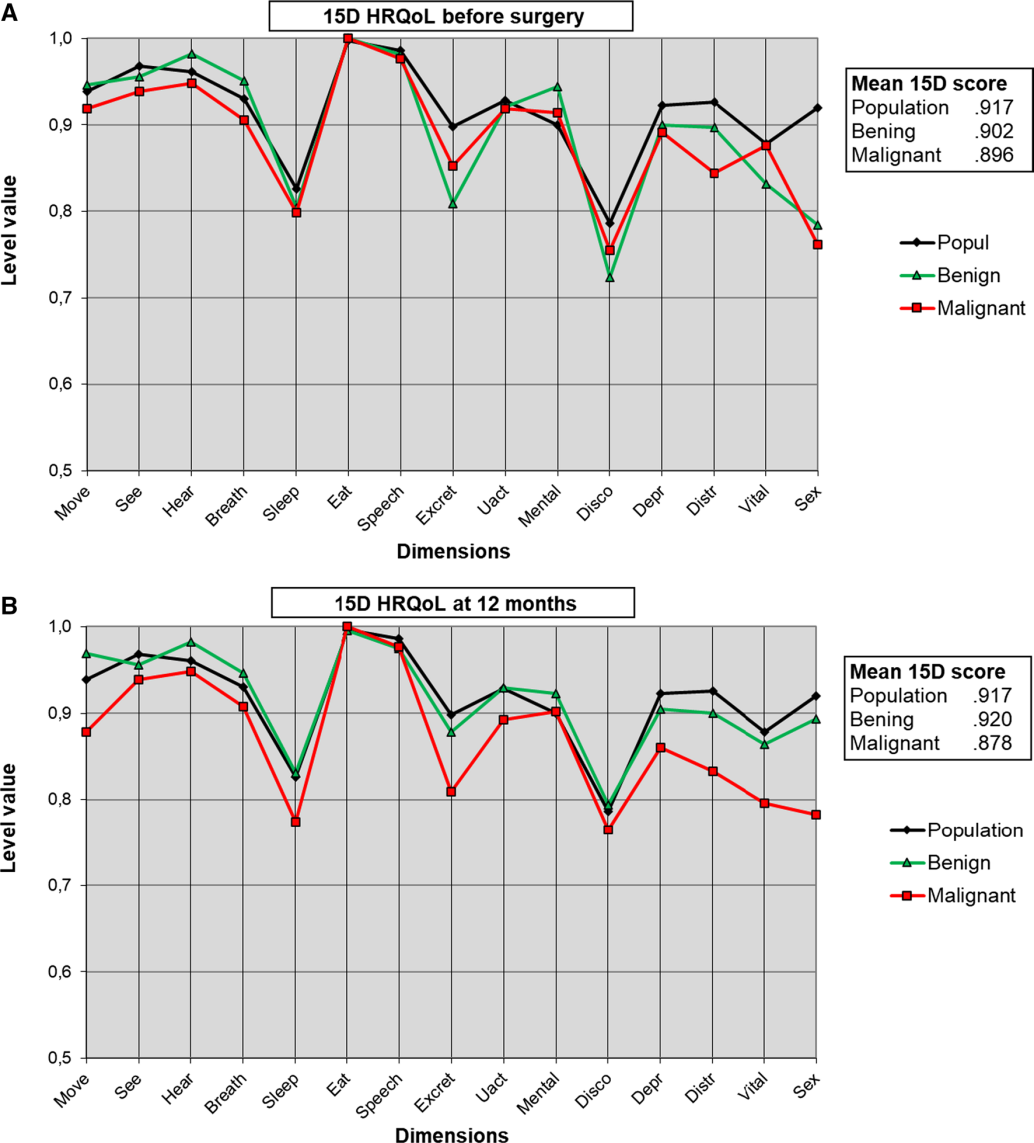
**A** and **B** Mean health-related quality of life before surgery and at 12 months after surgery. Move = moving, See = Seeing, Hear = Hearing, Breath = breathing, Sleep = sleeping, Eat = eating, Speech = speech, Excret = excretion, Uact = usual activities, Mental = mental function, Disco = discomfort and symptoms, Depr = depression, Distr = distress, Vital = vitality and Sex = sexual activity; Popul = age- and sex-matched control population (n = 2743); Malign = gynecologic surgery patients with malignant diseases; Benign = gynecologic surgery patients with benign diseases

The mean HRQoL score (15D) before surgery was similar in patients with benign (0.902) and malignant diseases (0.896, *p* = 0.640) but was lower than in the population (0.917, *p* = 0.017). At 12 months after surgery, the mean HRQoL score had increased in patients with benign diseases (0.920, *p* = 0.090) but decreased in patients with malignant diseases (0.878, *p* = 0.154). Therefore, at 12 months after surgery, the mean 15D score was lower in patients with malignant diseases (*p* = 0.020) and similar to that in the population in patients with benign diseases (*p* = 0.929). The difference between the two groups (0.042) was clinically important, as was the increase in patients with benign diseases (0.018) and the decrease in patients with malignant diseases (0.018). Patients with malignant diseases had more distress before surgery than those with benign diseases, and at 12 months their movement, distress, vitality and sexual activity scores were worse (Fig. 2A, B).

Resilience was moderately high in both groups, and no differences existed between the groups or time points. Dichotomized questionnaire scores were also similar between the groups (before surgery *p* = 0.920, at 12 months *p* = 0.783) (Table 4). Before surgery, resilience had a weak positive correlation with HRQoL (Pearson’s r = 0.187) and a moderate negative correlation with life satisfaction (r = − 0.360), anxiety (r = − 0.409), and depression (r = − 0.422). The correlation with life satisfaction was negative due to inverse scoring on the LS-4 scale; lower scores indicating higher life satisfaction. At 12 months, the correlations were similar but more prominent: resilience had a moderate positive correlation with HRQoL (r = 0.485) and a strong negative correlation with life satisfaction (r = − 0.545), anxiety (r = − 0.530), and depression (r = − 0.614).

Before surgery, weak to moderate correlations were observed between HRQoL and anxiety (r = − 0.263), depression (r = − 0.416), and life satisfaction (r = − 0.399). At 12 months after surgery, these correlations were more prominent; HRQoL had a strong negative correlation with anxiety (r = − 0.540), depression (r = − 0.624), and life satisfaction (r = − 0.528).

### Pain and analgesics use

The data on pain and analgesic use are listed in Table 3.

The prevalence (*p* = 0.749) and intensity (*p* = 0.789) of preoperative pain and pain at 12 months after surgery (*p* = 0.111 and *p* = 0.120, respectively) were similar in the two groups. However, pain decreased in women with benign diseases both at rest (*p* = 0.007) and during movement (*p* < 0.001) but not in women with malignant diseases (*p* = 0.259 at rest, *p* = 0.112 during movement). In patients with malignant diseases, preoperative pain (n = 34) persisted at 12 months after surgery in 20 (25%) patients, 10 (12%) had pain before surgery but not at follow-up, 17 (21%) had new pain one year after surgery, and 24 (30%) patients had no pain before or at 12 months after surgery. For patients with benign diseases, the numbers were 42 (22%) for persistent pain, 33 (17%) for pain resolution, 27 (14%) for new pain, and 65 (34%) patients for no pain before or at 12 months after surgery (*p* = 0.385 between groups).

The prevalence of persistent postoperative pain (PPOP) was similar in both groups; 18 out of 171 patients with benign diseases (11%), and 13 out of 71 patients with malignant diseases (18%, *p* = 0.099) had surgery-related pain at 12 months after surgery. In most patients with PPOP, the pain intensity was mild; four (13%) of them had moderate or severe pain (NRS 5–7) at rest, and three (10%) had moderate or severe pain during movement (NRS 5–8). The prevalence of moderate or severe pain was higher in patients with no PPOP at 12 months, 15 (20%) patients at rest (*p* = 0.401) and 13 (17%) patients during movement (*p* = 0.328).

Higher resilience scores before surgery correlated weakly positively with lower pain at rest (r = − 0.166) and during movement (r = − 0.170) and with a lower number of different analgesics in use (r = − 0.194) and regular pain medication (r = − 0.127) at 12 months after surgery. Higher resilience correlated weakly positively with better pain relief by analgesics before surgery (r = 0.290) and moderately positively at 12 months after surgery (r = 0.355).

Anxiety (r = 0.214) and depression (r = 0.239) before surgery correlated weakly positively with having pain one year after surgery.

The expected postoperative pain was similar between the two groups; patients with benign diseases expected to have the most postoperative pain score of 6.5 (2.3) and patients with malignant diseases 7.0 (1.9) (*p* = 0.099) on NRS 0–10. However, there was only a weak positive correlation between the expected and observed most pain score after surgery (r = 0.252). Patients with benign and malignant diseases were willing to accept similar pain before taking analgesics when asked before surgery, 4.4 (2.0) vs. 4.2 (2.1) (*p* = 0.357), and at 12 months after surgery, 6.2 (2.0) vs. 5.7 (2.4) (*p* = 0.172).

Before surgery, two-thirds (n = 169) and at 12 months after surgery, half (n = 114) of the patients used analgesics. There was no difference in preoperative pain medication use between the two groups (*p* = 0.089), but at 12 months after surgery, 40 patients with malignant diseases (56%) used analgesics more commonly than 74 patients with benign diseases (43%) (*p* = 0.018).

Opioid analgesic use was low; at 12 months, there were eight (3%) new patients on opioids, eleven (5%) had stopped opioid use, opioid use persisted in four patients (2%), and 219 (90%) patients used opioids neither before nor at 12 months after surgery (*p* < 0.001).

The groups did not differ in pain relief achieved by analgesics before surgery (*p* = 0.540), but one year after surgery, patients with malignant diseases experienced better pain relief than those with benign diseases (*p* = 0.004) (Table 3).

Pain medication-related adverse effects before surgery (n = 18) and at 12 months (n = 22) were relatively rare. The most common adverse effects were gastrointestinal and comprised constipation, stomachache, and diarrhea.

### Other outcome measures

The anxiety and depression scores are listed in Table 4. Before surgery, patients with malignant diseases had more anxiety (*p* = 0.008). Anxiety decreased during the 12-month follow-up in both groups, and at 12 months, there were no differences between the two groups (*p* = 0.972). Depression scores were similarly low in both groups and at both time points.

Life satisfaction was similarly high in both groups (Table 4), but patients with malignant diseases had lower life satisfaction before surgery (*p* = 0.011). Life satisfaction increased in patients with malignant diseases (*p* = 0.023) but not in patients with benign diseases (*p* = 0.249) and was thus similar in the two groups at 12 months after surgery (*p* = 0.335).

A post hoc sensitivity analysis showed that patients lost to follow-up (n = 29) had similar mean preoperative questionnaire scores to other patients. No difference existed in HRQoL (*p* = 0.457), resilience (*p* = 0.755), anxiety (*p* = 0.240), depression (*p* = 0.277), or life satisfaction scores (*p* = 0.06) between the respondents and non-respondents.

## Discussion

The novelty of this study was that we evaluated resilience for the first time in gynecology patients undergoing surgery for benign and malignant diagnoses. In the present study, resilience was moderately high, and patients with malignant diseases had comparable resilience scores to previous studies in gynecological cancer [12, 15]. In the present study, HRQoL decreased in patients with malignant diseases and increased in those with benign diseases. Thus, in contrast to those before surgery, patients with malignant diseases had clinically importantly lower HRQoL than those with benign diseases at 12 months after surgery. Consistent with previous studies [7, 11, 12, 15, 28], higher resilience had a moderate to strong correlation with better HRQoL, higher life satisfaction, and lower anxiety and depression. Anxiety decreased in both groups during the follow-up, but depression was rare before and after surgery. Our study concurred with our hypothesis on pain and pain relief, as higher resilience correlated with less pain one year after surgery, less analgesic use, and better pain relief achieved by pain medication.

In our study, one out of four patients had low resilience, RS-25 score ≤ 130/175, which is consistent with previous studies about resilience in gynecological cancer [12, 15] and breast cancer patients [13]. The means of the resilience scores were moderate, 142–143, in both cohorts and were similar to a Nordic population study [29] of women between 19 and 89 years old. Resilience is not just an inherited trait but can also be developed with time. Furthermore, promising research results exist for improving resilience in the clinical setting [30, 31]. Because more resilient patients had better pain and HRQoL outcomes after gynecological surgery, we propose that in preoperative evaluation, resilience should be evaluated, and interventions to enhance resilience, counseling, and close follow-up should be focused on patients with low resilience.

Before surgery, the HRQoL of all patients was clinically importantly worse than that of the age-matched general female population, but at 12 months, the total scores were similar in patients with benign diseases to those of the population, but in patients with malignant diseases, their HRQoL was substantially worse. Before surgery, patients scored lower than the general population on five dimensions of the 15D: excretion, depression, distress, vitality, and sexual activity. At 12 months, dimensions of moving and vitality had decreased in patients with malignant diagnoses and conversely improved in patients with benign diagnoses on the dimensions of excretion, sexual activity, and discomfort and symptoms. The 15D dimension improvements in patients with benign diseases are consistent with previous studies of women undergoing hysterectomy [2] and pelvic organ prolapse surgery [18]. Both hysterectomy and prolapse surgery were also common procedures in the present study. Future studies should consider subgrouping patients with malignant diseases according to neoadjuvant and adjuvant therapy due to the impact on symptoms and quality of life.

In accordance with previous research, patients with malignant diseases had more anxiety than those with benign diseases before surgery, and anxiety decreased after surgery in both benign and malignant diagnoses. Anxiety and depression scores in our study in patients with malignant diseases were similar to those in another European study [3]. In our study, the mean HADS anxiety score (0–21) was 7.0, and the mean HADS depression score (0–21) 3.4 compared to 7.1 and 3.5 in the Ferrandina et al. study [3], respectively. Similar scores have been reported in other studies [5, 32], but the prevalence of depressive symptoms varies substantially between countries [33–35]. Cultural aspects seem to affect psychological symptoms observed in women undergoing gynecological surgery [36]. In the present study, in patients with malignant diseases, severe anxiety was fourfold more common (16%) than in the Finnish general population (4%), but it decreased to population levels (4%) at 12 months after surgery [37]. For benign diagnoses, the prevalences were 9% and 6%.

In the present study, the prevalence of depression was lower than in the general population [38], which is consistent with a study by Kim et al. [34]. Before surgery none of the women with malignant diseases had severe depression and at 12 months, only two (3%) had severe depression, which is substantially less than that in the general population (5–7%). Severe depression was also uncommon in patients with benign diseases; before surgery, four (2%), and at 12 months, three (2%) patients had HADS depression scores ≥ 11/21. In other words, the course of psychological symptoms during the follow-up was supported by the current literature [3, 5, 32], but depression symptoms were lower than expected in both groups. This supports our hypothesis that the majority of patients were resilient, which helped them adjust and minimize their psychological symptoms and sustain or increase their life satisfaction.

Persistent postoperative pain is an issue. For two decades, PPOP has been recognized as one of the major adverse events after surgery. In a survey in 1998, Crombie and colleagues [39] found that pain after surgery was the second most common reason for seeking care in pain centers and that in these patients, pain was particularly associated with abdominal, anal, perineal, and genital areas. They found that PPOP was more common in women than in men [39]. In the present study, the prevalence of PPOP was 13%, and it was slightly more common in patients with malignant diseases than in those with benign diseases. The prevalence of PPOP was similar to that reported in patients undergoing robot-assisted laparoscopic hysterectomy [40]. As chronic pain is often reported by women after gynecologic surgery, Brandsborg and colleagues have proposed that in the preoperative assessment, patients’ coping strategies should be evaluated and that a postoperative assessment should be done a few months after surgery to identify those at risk of developing PPOP [41].

Another issue is the safety of opioid use in surgical patients, as some studies have indicated that the incidence of new persistent opioid use is relatively common even after minor surgical procedures [42, 43]. In the present study, all but four patients used perioperative opioid analgesics, 6% of patients used opioid analgesics before surgery, and 5% used them at 12 months after surgery. At 12 months, there were three new persistent opioid users, and eleven had stopped preoperative use. Thus, it seems that in Finland, perioperative opioid use is not a risk for persistent use of opioid analgesics. In Finland multimodal postoperative pain management is a clinical standard, and oxycodone has been a commonly used opioid since the early 1960s. Based on this expertise and awareness on addiction and misuse potential, postoperative pain management is based on nonopioid analgesics, and oxycodone or other opioids are prescribed only for severe pain not relieved with other options [44].

### Limitations

This study acknowledges its limitations. Despite the proper total sample size (n = 271), the benign and malignant diagnostic groups differed significantly in size (190 vs. 81), and both included multiple different diagnoses that led to gynecologic surgery. Moreover, patients with malignant diagnoses were not grouped based on adjuvant therapy, nor did we account for neoadjuvant therapy, which can impact symptoms and quality of life. Consequently, these results cannot be generalized to specific diagnoses. Nonetheless, the purpose of this study was to form a general concept and compare resilience, pain, and HRQoL in non-selected groups of benign and malignant gynecological diagnoses.

Second, the questionnaires were self-administered, and individuals could interpret the survey questions differently. Nevertheless, the questionnaires in our study have been validated and are commonly used. We assumed that questionnaires with closed-end questions could be easier for individuals to answer, especially when evaluating sensitive issues. Interviewer bias is minimized in self-report questionnaires, and this approach is thus less likely to have the Hawthorne effect. The questionnaires were web-based, which can make older people more reluctant to answer, but paper copies were available on request. Adherence to questionnaires and the participation rate to the follow-up were high and decreased the possibility of compliance bias. Moreover, the post hoc analysis found similar characteristics between respondents and non-respondents in the preoperative assessment.

Third, we measured pain with no pain interference measures. Previous studies showed that in patients with low resilience, pain has more interference with activities of daily living and life satisfaction than in more resilient patients [16, 45, 46]. Our study’s approach can give an oversimplified image of the multiple effects of pain. However, our results are supported by the existing literature.

Finally, our study had multiple variables that increases a potential for researcher bias. Postoperative pain has a multifactorial origin and associates with pre-operative patient conditions, surgical procedure, duration of surgery, and postoperative analgesic therapy. In the present study laparotomy was used more commonly in women with malignant diseases and none had vaginal surgery, compared to women with benign diseases having only a few laparotomies and one-third having vaginal surgery, moreover the duration of surgery was two times longer in women with malignant diseases compered to women with benign diseases. Minimally invasive techniques and vaginal approach are assumed to reduce postoperative pain, and the need of analgesics [47], but the evidence is vague. Early pain after laparoscopic surgery can be more severe than that after open approach [48]. However, were analyzed our data based on our analysis plan and hypothesis. We performed multiple analyses and reported the results consistently, focused on the main hypotheses, and the findings were in accordance with previous research. Consequently, we believe that our data and analyses are sound.

## Conclusions

In our study, resilience was moderately high in the majority of gynecology patients undergoing surgery for benign or malignant diagnoses, but one out of four patients reported low resilience. The HRQoL of all patients was lower than that of the general population before surgery, but at 12 months, it increased in patients with benign diseases and was therefore similar to that of the age-matched general female population. Higher resilience correlated with better HRQoL and less pain 12 months after surgery. Patients with low resilience should be identified during preoperative evaluation, and health care professionals should give these patients psychological support to enhance their resilience.

## Data Availability

The datasets generated and analysed during the current study are not publicly available due to potentially identifying data and sensitive patient information but are available from the corresponding author on reasonable request.

## Abbreviations

15D: The 15D instrument of health-related quality of life
EMRs: Electronic medical records
HADS: Hospital Anxiety and Depression Scale
HRQoL: Health-related quality of life
KUH: Kuopio University Hospital
LS-4: Life Satisfaction scale-4
NRS: Numeral rating scale
PACU: Postanesthesia care unit
PPOP: Persistent postoperative pain
RS-25: Resilience scale-25
